# Supplementary material for: An Exogenous Pre-Storage Melatonin Alleviates Chilling Injury in Some Mango Fruit Cultivars, by Acting on the Enzymatic and Non-Enzymatic Antioxidant System
Source: Antioxidants (Basel). 2022 Feb 14;11(2):384. doi: 10.3390/antiox11020384 (PMC8869158; doi:10.3390/antiox11020384)
Supplement: Supplementary file 1 [file antioxidants-11-00384-s001.zip › antioxidants-1588481-supplementary.pdf]

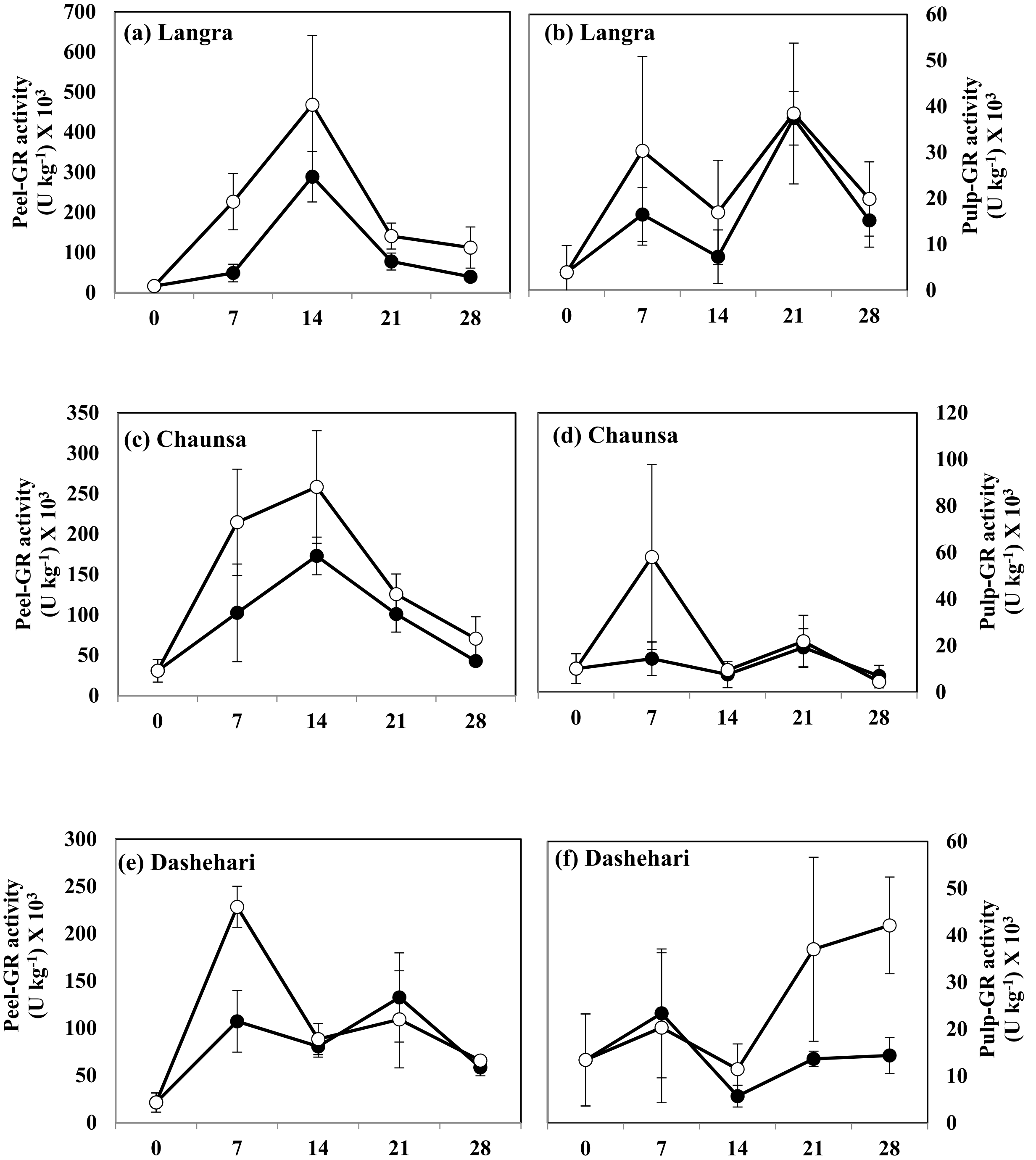

(a) Langra
(b) Langra
(c) Chaunsa
(d) Chaunsa
(e) Dashehari
(f) Dashehari
Peel-GR activity (U kg-1) X 103
Pulp-GR activity (U kg-1) X 103
0
7
14
21
28

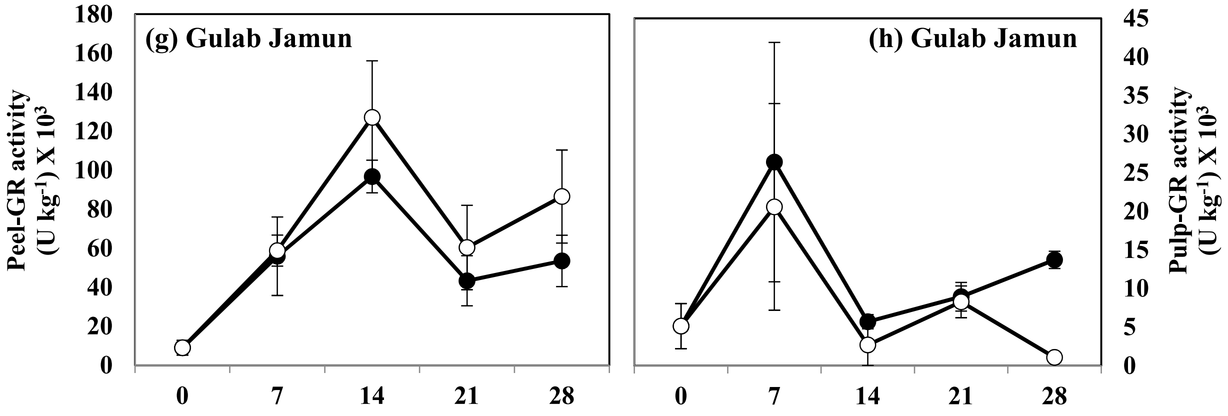

Figure. S1. Glutathione reductase (GR) activity in **(a)** 'Langra' peel, **(b)** 'Langra' pulp, **(c)** 'Chaunsa' peel, **(d)** 'Chaunsa' pulp, **(e)** 'Dashehari' peel, **(f)** 'Dashehari' pulp, **(g)** 'Gulab Jamun' peel, and **(h)** 'Gulab Jamun' pulp treated with 0 μM (control) or 100 μM (treated) MT for 2 h, followed by 28 d of low temperature storage (5 ± 1 ºC) and 3 d of shelf life at room temperature. Measurements were taken every 7 d of storage. Each value is the mean of three replicates ± standard error. An asterisk (*) on the same storage period indicates significant differences ($p < 0.05$).

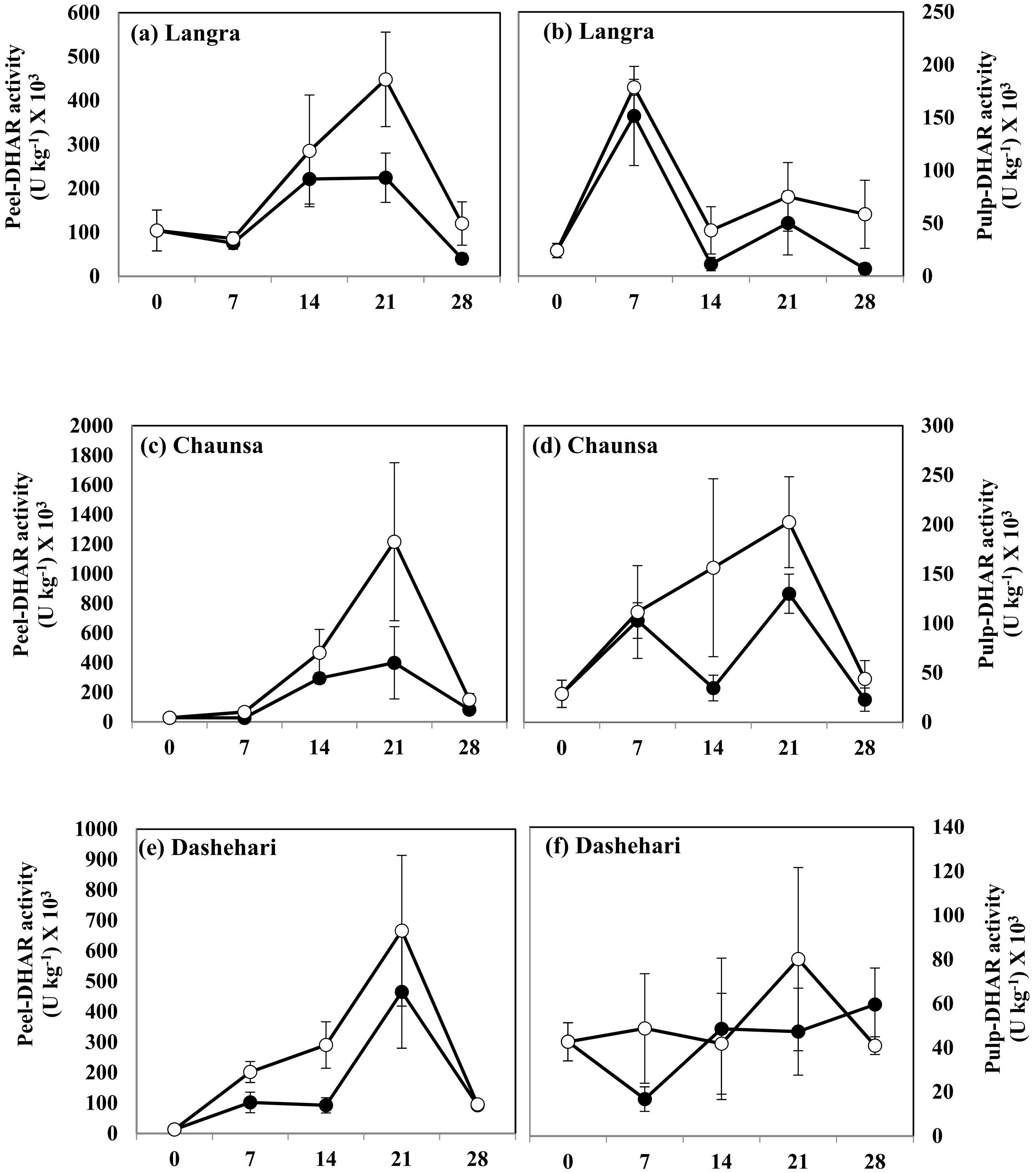

(a) Langra
(b) Langra
(c) Chaunsa
(d) Chaunsa
(e) Dashehari
(f) Dashehari
Peel-DHAR activity (U kg^{-1}) X 10^3
Pulp-DHAR activity (U kg^{-1}) X 10^3
0
7
14
21
28

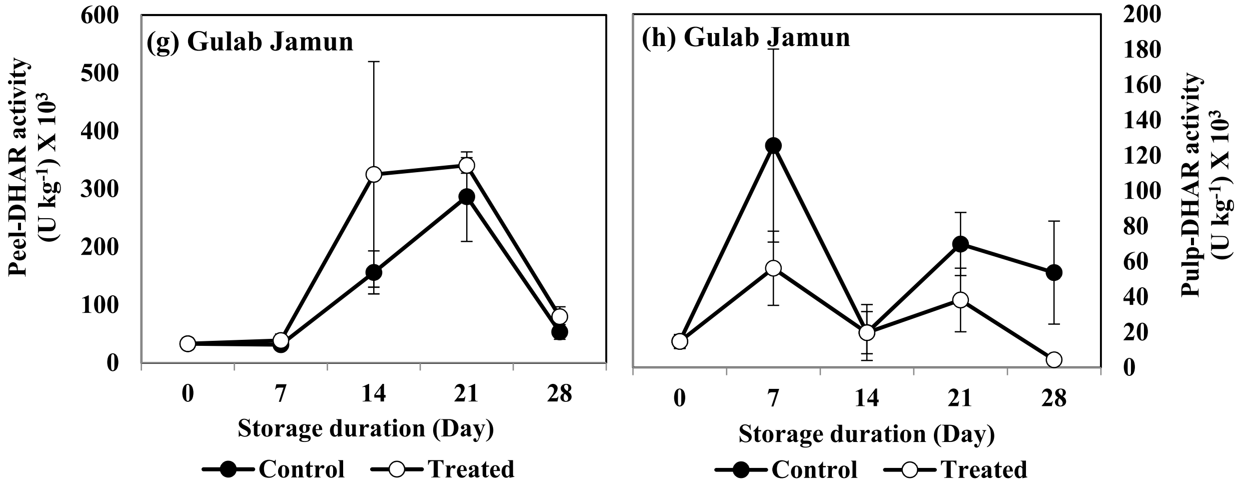

Figure. S2. Dehydroascorbate reductase (DHAR) activity in **(a)** 'Langra' peel, **(b)** 'Langra' pulp, **(c)** 'Chaunsa' peel, **(d)** 'Chaunsa' pulp, **(e)** 'Dashehari' peel, **(f)** 'Dashehari' pulp, **(g)** 'Gulab Jamun' peel, and **(h)** 'Gulab Jamun' pulp treated with 0 µM (control) or 100 µM (treated) MT for 2 h, followed by 28 d of low temperature storage (5 ± 1 ºC) and 3 d of shelf life at room temperature. Measurements were taken every 7 d of storage. Each value is the mean of three replicates ± standard error. An asterisk (*) on the same storage period indicates significant differences ($p < 0.05$).
